# Towards an Efficient One-Class Classifier for Mobile Devices and Wearable Sensors on the Context of Personal Risk Detection

**DOI:** 10.3390/s18092857

**Published:** 2018-08-30

**Authors:** Luis A. Trejo, Ari Yair Barrera-Animas

**Affiliations:** Tecnologico de Monterrey, Escuela de Ingeniería y Ciencias, Carretera al Lago de Guadalupe Km. 3.5, Atizapán, Edo. de México C.P. 52926, Mexico; ybarrera@itesm.mx

**Keywords:** time-domain features, frequency-domain features, principal component analysis, behaviour analysis, classifier efficiency, personal risk detection, one-class classification, wearable sensors

## Abstract

In this work, we present a first step towards an efficient one-class classifier well suited for mobile devices to be implemented as part of a user application coupled with wearable sensors in the context of personal risk detection. We compared one-class Support Vector Machine (ocSVM) and OCKRA (One-Class K-means with Randomly-projected features Algorithm). Both classifiers were tested using four versions of the publicly available PRIDE (Personal RIsk DEtection) dataset. The first version is the original PRIDE dataset, which is based only on time-domain features. We created a second version that is simply an extension of the original dataset with new attributes in the frequency domain. The other two datasets are a subset of these two versions, after a feature selection procedure based on a correlation matrix analysis followed by a Principal Component Analysis. All experiments were focused on the performance of the classifiers as well as on the execution time during the training and classification processes. Therefore, our goal in this work is twofold: we aim at reducing execution time but at the same time maintaining a good classification performance. Our results show that OCKRA achieved on average, 89.1% of Area Under the Curve (AUC) using the full set of features and 83.7% when trained using a subset of them. Furthermore, regarding execution time, OCKRA reports in the best case a 33.1% gain when using a subset of the feature vector, instead of the full set of features. These results are better than those reported by ocSVM, in which case, even though the AUCs are very close to each other, execution times are significantly higher in all cases, for example, more than 20 h versus less than an hour in the worst-case scenario. Having in mind the trade-off between classification performance and efficiency, our results support the choice of OCKRA as our best candidate so far for a mobile implementation where less processing and memory resources are at hand. OCKRA reports a very encouraging speed-up without sacrificing the classifier performance when using the PRIDE dataset based only on time-domain attributes after a feature selection procedure.

## 1. Introduction

It is highly desirable to recognise as soon as possible whenever a person faces a risk-prone situation that could threaten that person’s physical integrity. Barrera-Animas et al. in [1] introduce the term *Personal Risk Detection* as an attractive line of research focused on this timely detection. Their hypothesis is based on the observation that people express regular patterns, with small variations, regarding their normal physical and behavioural activities. These patterns would change, even drastically, whenever the person is facing a hazardous situation. A wearable device with a set of simple and common sensors, such as heart rate, accelerometer, gyroscope, and skin temperature, just to mention a few, is sufficient to capture deviations from normal behaviour. Personal risk detection can be tackled as an anomaly detection problem that aims at differentiating a normal condition from unusual behaviour. In this context, the use of one-class classification algorithms has shown very good results. Actually, Barrera-Animas et al. reported in [1] that one-class Support Vector Machine (ocSVM) achieved the best performance among the classifiers used in their experiments. Later on, a new one-class classifier named One-Class K-means with Randomly-projected features Algorithm (OCKRA) proposed by Rodríguez et al. in [2] was introduced for the personal risk detection problem. In their research, they showed that OCKRA achieved the best results in the classification task, leaving ocSVM in second place.

As stated in [1], Vital Signs Monitoring (VSM) and Human Activity Recognition (HAR) fields are closely related to the personal risk detection problem. From research works presented in both fields, a trend to use features in frequency and time domains can be noticed [3,4,5,6]. The rationale behind this approach lies in the nature of recorded sensors measurements and that their treatment in frequency-domain reveals several features that can not be appreciated in the time-domain. Thus, the inclusion of features in both domains generally gives a more complex and complete view of the observed scenario. Furthermore, in HAR and VSM research fields, several works pursue the use of distinct specialised sensors to gather the most possible information about individuals and their environment. For instance, an interesting review work on this subject is that of Rucco et al. [7], which describes the state-of-the-art research on fall risk assessment, fall prevention and detection. Their review surveys the most adopted sensor technologies used in this field and their position on the human body, with special interest in healthy elderly people. With this approach, it is also intended to increase the number of features obtained that characterise the study case.

Advantages such as to gain a better understanding of the physiological and behavioural patterns of an individual, and to avoid lack of information and data concurrency, result from increasing the number of used sensors and features. However, despite the fact that using several sensors and deriving diverse features in time/frequency-domain has some advantages, an important issue could arise from this approach: a high dimensionality problem. Reducing dimensionality comprises the process of project high-dimensional data into a low-dimensional space while retaining its variability [8]—that is, to reveal a relevant low-dimensional space embraced in the high-dimensional one [9]. Principal Component Analysis (PCA) is one of the classic techniques used in the literature to reduce the dimensionality of a dataset due to its capability to reveal the hidden structure of data, even on high-dimensional space, and to its low computational consumption requirements [9]. Regarding the personal risk detection problem, to the authors’ knowledge, there is no research work based on this approach. However, several research studies across multiple disciplines integrate frequency-/time-domain features and deal with the dimensionality reduction problem, as we briefly describe in Section 2.

The aim of the present research is twofold. Firstly, we are interested in exploring the impact of using dimension reduction techniques and frequency domain features in the context of the personal risk detection problem. We use a correlation matrix and Principal Component Analysis for the dimension reduction task as they are well studied and implemented in related research concerning classification problems. The second aim is to speed-up the training and classification process of a given classifier, without sacrificing its performance. This is a very important requirement since our final classifier is meant to be implemented on mobile devices; thus, efficiency is paramount due to a limitation on memory and CPU resources.

The rest of the document is organised as follows: in Section 2, we briefly review recent work that is closely related to ours; in Section 3, we present the PRIDE dataset used in our experiments, the pre-processing of PRIDE and feature selection methodology in the time and frequency domains. Additionally, the one-class classifiers used in our experiments are introduced. Next, in Section 4, we present our experimental results and, to reinforce their validity, we discuss the outcome of statistical tests run over our algorithms. Finally, in Section 5, we give our conclusions about the outcomes of this work and ideas for current and future work.

## 2. Related Work

Pei et al. [3] work focused on three main topics: context sensing, modelling human behaviour, and the development of a new architecture intended for a cognitive phone platform. Time and frequency domain features were comprised in their study. Furthermore, a sequential forward selection algorithm was used during the feature selection process carried out before training any classifier. Test results showed an accuracy rate up to 92.9%.

Özdemir and Barshan [4] used an accelerometer, gyroscope, and magnetometer/compass tri-axial sensors to detect people’s falls by means of six wearable sensors. They set up a controlled environment to capture data when falling occurs. In their work, they derived a 1404-dimensional feature vector, using variables in the time and frequency domains; later, they employed a Discrete Fourier Transform. Afterwards, PCA was used to reduce the feature vector high dimensionality and complexity in training and testing the classifiers, obtaining a 30-dimensional feature vector. As classifiers, they used Least Squared Method, k-Nearest Neighbour, Support Vector Machines, Bayesian Decision Making, Dynamic Time Warping, and Artificial Neural Networks. Furthermore, they computed the computational cost for training and testing of each classifier for a single fold. Results showed an accuracy over 95% for the six tested classifiers. The authors conclude that k-Nearest Neighbour and Least Squared Method are suitable for real-time applications since their computational requirements are acceptable in the training and testing phase.

Wundersitz et al. [5] research was centred on the classification of team sport-related activities using data obtained from accelerometers and gyroscopes. In their study, frequency and time domain features were calculated and used to train different classifiers. Frequency-domain features were calculated via the fast Fourier transformation. Moreover, ANOVA (Analysis of variance) and LASSO (Least absolute shrinkage and selection operator) regression analysis were used as feature selection methods to reduce the processing time and to make the model easier to interpret. As part of their conclusions, they stated that it is possible to reduce the processing time through feature selection, but decreasing the classification accuracy. However, they also concluded that further exploration of features and feature selection is needed.

Lian [10] showed that PCA can be used as a dimension reduction tool for an ocSVM classifier with good results. His research takes as a baseline one of the most popular dimension reduction tools used in unsupervised and supervised problems, PCA. However, instead of extracting eigenvectors associated with top eigenvalues, he extracts the eigenvectors associated with small eigenvalues. In this approach, the null of the eigenspace is of interest since common features contained in the training samples are described by the null space.

Su et al. [11] work explores the dimension reduction of a hyperspectral images (HSI) dataset through feature selection and feature extraction techniques. Their goal was to augment the classification accuracy obtained by SVM. To reduce the size of the training dataset, they tested the following algorithms: mutual information, minimal redundancy maximal relevance, PCA, and Kernel PCA. Their experiments were centred on the performance achieved by SVM using the number of features selected by each technique. Results showed that PCA was the most effective technique to reduce data dimensionality in terms of computational load, implementation complexity, and classification performance. Furthermore, they showed that using SVM in combination with PCA obtains better prediction performance in terms of accuracy than using SVM with the full dataset. The authors concluded that using SVM with PCA is suitable for real-time applications since there is a significant reduction in computational time.

As we have seen in the reviewed work, a common approach is to work with attributes in both domains, time and frequency, and then apply feature selection techniques before training any classifier. Following this approach, it is possible to minimise the number of computed features and thus reduce the processing time required to train the classifiers, but, at the same time, trying to keep a good classification performance.

## 3. Methods

In this section, we describe in detail the datasets used in our experiments as well as the feature selection procedures performed on the datasets. In addition, we describe the classifiers used to compare the efficiency of the feature selection process.

### 3.1. PRIDE Dataset

Barrera-Animas et al. [1] built a new dataset specifically oriented to the personal risk detection problem, so that the research community could use it for a fair comparison of algorithms. The dataset is known as PRIDE and contains sensor data from 23 subjects wearing a Microsoft Band v1^©^ (Microsoft Corporation, Washington, DC, USA), and by means of a mobile application developed by the authors, they collected sensor data from the band, and uploaded it to an FTP (File Transfer Protocol) server. This procedure was done during one week, 24 h a day, to create the normal conditions dataset (NCDS), which is part of PRIDE. During this period, subjects made sure that their week was an ordinary one. PRIDE includes subjects with diverse individualities regarding gender, age, height and lifestyle. The dataset comprises 15 male and eight female volunteers aged in the range 21 to 52 years, statures from 1.56 to 1.86 m, weights in the range 42 to 101 kg, exercising practice of 0 to 10 h per week, and sedentary hours or leisure ranging from 20 to 84 h per week. Afterwards, to build the PRIDE’s anomaly conditions dataset (ACDS), the same 23 subjects participated in another process to obtain data under specific conditions, for which five scenarios to simulate hazardous or abnormal conditions were designed. These scenarios involved the following activities: running 100 m as fast as possible, climbing the stairs in a multi-floor building as quick as possible, a two-minute boxing episode, falling back and forth, and holding one’s breath for as long as possible. Each activity intended to simulate anomalous conditions, comprising possibly risk-prone situations from real world, e.g., running away from an unsafe situation, clearing a building due to an emergency alert, defending against an aggressor during a dispute, swooning and suffering from breathing problems. The session to perform all five scenarios by each subject lasted for about two hours, and it demanded major physical effort. They were realised indoors and outdoors with different weather conditions and levels of UV exposure, depending on the day the subject was able to present them. The elderly, and other groups such as people suffering a chronicle disease, comprise very important groups in our society; however, they were not included in the data collection process, due to the demanding nature of the method just described.

As mentioned previously, personal risk detection can be approached as an anomaly detection problem, to differentiate a normal condition from uncommon behaviour. The anomaly detector can be a one-class classifier, trained only with a user’s normal conditions dataset. The stress scenarios serve to verify if the classifier is able to distinguish them as an anomaly, and not to recognise which scenario or activity is being observed. The stress scenarios are intended to simulate certain danger or abnormal behaviour; however, we acknowledge that they are only an estimate to real-life situations. Our goal is to detect anomalies that can be the result of, for example, a car accident, a health crisis, or a physical aggression. We decided to undertake this approach since we do not have the means to obtain data in the course of a real-life crisis. It is worth remarking that anomalous situations in some cases may be related to a personal risk-prone situation. However, labelling a behaviour as abnormal does not always imply risk; moreover, not all risk-prone situations will always turn into anomalous behaviour. In other words, we are able to differentiate abnormal behaviour from ordinary one, thereby spotting some (but not all) possible risk-prone circumstances.

Next, we briefly describe the sensors embedded in the wearable device. Table 1 lists the sensors in the band and their operating frequencies; using these values, a user gathered on average 1.6 millions of records per day. A readout from the accelerometer and gyroscope was obtained every 125 ms, using an operating frequency of 8 Hz. Ultraviolet exposure values are gathered every 1 min and skin temperature values every half a minute. The measurements of the rest of the band’s sensors, distance, pedometer, heart rate, and calories are logged every 1 s.

In the following sections, we present our methodology for feature selection in time and frequency domains, respectively. By running the Kruskal–Wallis test, we proved that all users’ datasets are statistically different, that is, they are not drawn from the same population. Hence, we decided to use a subject-dependent approach during the feature selection process, i.e., on a user-by-user basis. It is important to notice that the feature selection process is performed only on the user’s training dataset, that is, the Normal Conditions Dataset (NCDS).

### 3.2. PRIDE Pre-Processing and Feature Selection in the Time-Domain

During the pre-processing step, a feature vector is computed using windows of one second of sensor data; this process is done for every user in the PRIDE dataset. Depending on the readout interval of a sensor, three rules apply in order to assign a value to the feature vector:

$$\mathrm{Feature}\;\mathrm{vector}=\left\{\begin{array}{cc} \mathrm{Average}\;\mathrm{and}\;\mathrm{sample}\;\mathrm{standard}\;\mathrm{deviation} & \\ \mathrm{of}\;\mathrm{all}\;\mathrm{sensor}\;\mathrm{measurements}\;\mathrm{in}\;a\;\mathrm{second}, & \;\mathrm{if}\;\;\mathrm{readout}\;\mathrm{interval}\;<\;1\;s,\\ \mathrm{Assign}\;\mathrm{the}\;\mathrm{sensor}\;\mathrm{value}, & \;\mathrm{if}\;\;\mathrm{readout}\;\mathrm{interval}\;=\;1\;s,\\ \mathrm{Assign}\;\mathrm{the}\;\mathrm{last}\;\mathrm{sensor}\;\mathrm{value}, & \;\mathrm{if}\;\;\mathrm{readout}\;\mathrm{interval}\;>\;1\;s. \end{array}\right.$$

Thus, a feature vector from a given window contains the following sensor values:

- Means and standard deviations of the gyroscope and accelerometer readouts.
- Absolute values from the heart rate, skin temperature, pace, speed, and ultraviolet exposure sensors.
- A Δ-value, computed as the difference between the current and previous values of the following measurements: total steps, total distance, and calories burned.

Using this procedure, a 26-dimensional feature vector is derived; its final structure is shown in Table 2. This feature vector in the time-domain was used to obtain all results reported in [1,2,12]. The subsets NCDS and ACDS of PRIDE are pre-processed using this procedure.

Finally, each of the user logs was divided into five folds to use them in a five-fold cross-validation. In the cross-validation, four folds of the normal behaviour of a user were used for training and one fold was joined with the anomaly dataset log to test the classifiers. This procedure was repeated five times alternating the user’s fold that was retained for testing. Hence, five training datasets and five testing datasets were set.

After this pre-processing step, we have conducted a Principal Component and a Correlation Matrix (CM) analysis on the PRIDE time-domain dataset with the aim of reducing its dimensionality. PCA allows identifying those features that best describe the variability of the points in the dataset, whereas the correlation matrix performs a statistical correlation analysis that is often used to remove redundant (highly-correlated) features [6,13,14,15,16].

**Figure 1:**
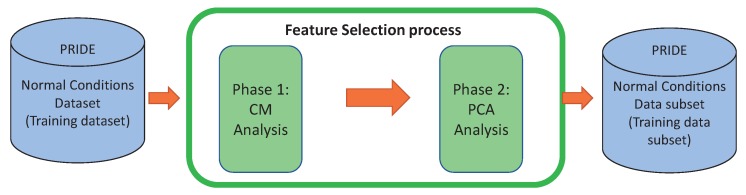
Feature selection process performed over every user of the PRIDE training dataset.

The experiments were conducted in R language, in the RStudio software (version:1.0.153 , RStudio Inc., Boston, MA, USA) [17]. The correlation matrix was computed using the well-known caret package [18]. Firstly, we performed the CM process to remove redundant features and then we applied PCA to remove features that do not contribute sufficiently to the principal components but at the same time retaining at least 60% of data variability [13,14,19]. This process is illustrated in Figure 1.

#### 3.2.1. Correlation Matrix Analysis on the Time-Domain Attributes

**Figure 2:**
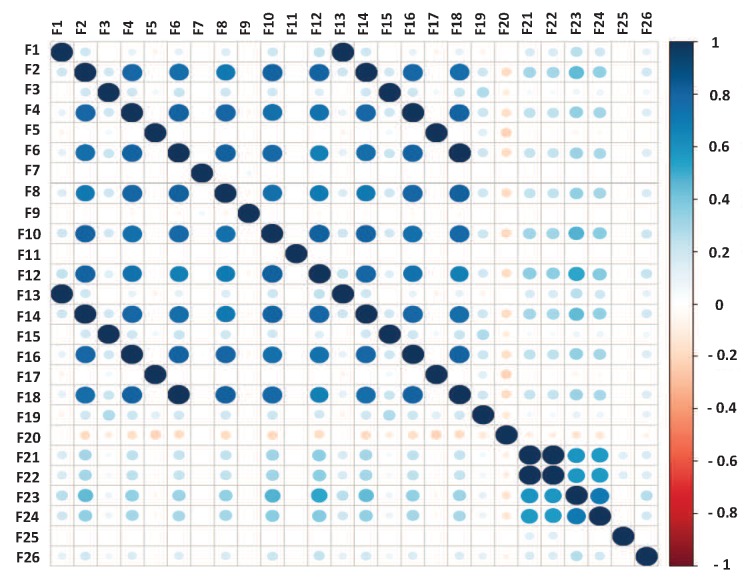
Correlation matrix of user 1.

We computed the correlation matrix for the 23 users in PRIDE. As a sample, the result for user 1 is shown in Figure 2. For each user, features with a correlation value equal to or greater than 0.75 are saved into a vector [20]. Next, we computed the frequency of occurrence of each feature along the 23 vectors. If a feature is reported as highly-correlated by at least 22 of the 23 vectors, then that feature is removed from the dataset.

The results in Table 3 show that features F2, F6, F10, F14, and F18 are highly-correlated consistently in at least 22 users, thus they were removed from the PRIDE dataset. Using the correlation matrix analysis for feature selection, the PRIDE dataset was downsized from 26 to 21 features.

#### 3.2.2. Principal Component Analysis on the Time-Domain Attributes

We run a Principal Component Analysis over every user of the PRIDE dataset in order to identify those features that best describe the variability of the data in the dataset; in this way, we are able to remove those features that do not contribute sufficiently to data variability.

**Figure 3:**
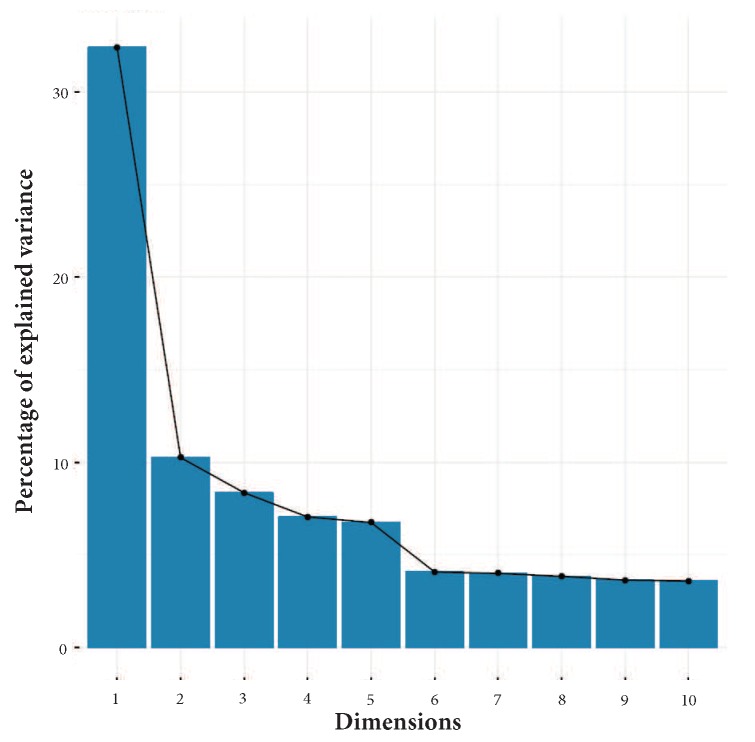
PCA results for user 1 from PRIDE that shows the percentage of explained variance of the first 10 dimensions.

**Figure 4:**
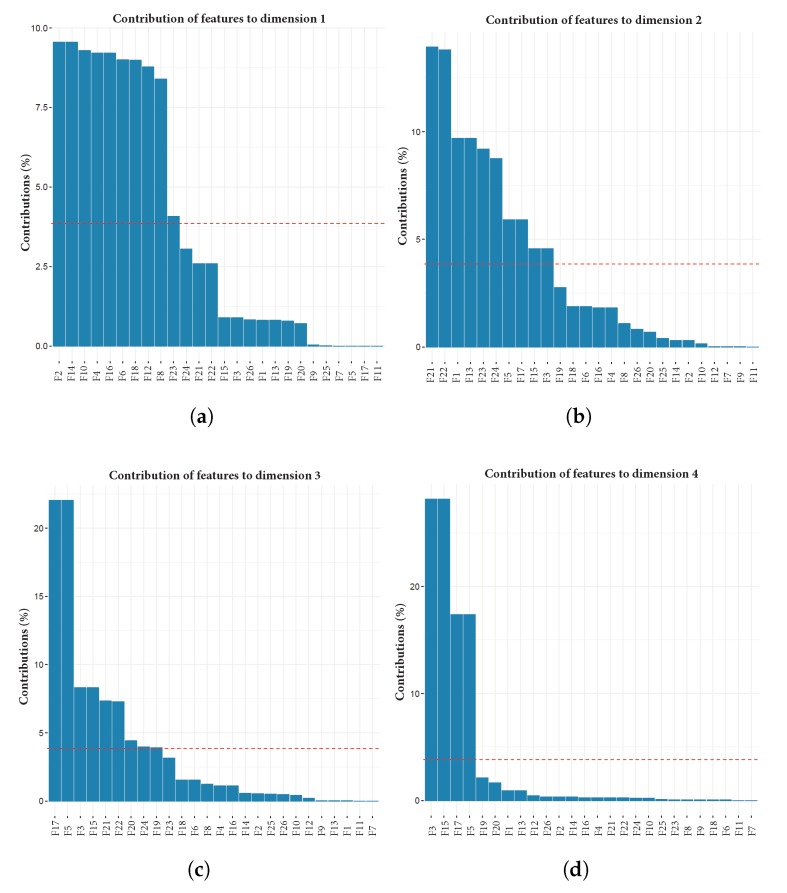

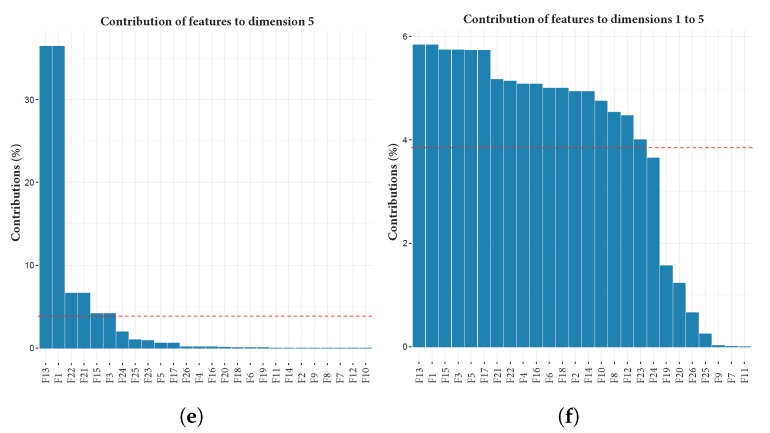
PCA results for user 1. Each graph represents the contribution of every feature to data variability in (**a**) dimension 1; (**b**) dimension 2; (**c**) dimension 3; (**d**) dimension 4; and (**e**) dimension 5; (**f**) contribution of every feature in the aggregated five dimensions.

Figure 3 shows the results of PCA computed over the PRIDE user with more data, user 1. It shows the percentage of the explained variances across ten dimensions. It can be observed, by aggregating the contribution of each dimension, that the first five dimensions explain approximately 60% of data variability [13,14,19]. Figure 4a–e depict a plot for each of the first five dimensions, with contribution percentage per variable in such dimension. Additionally, Figure 4f shows the contribution percentage of each variable to the aggregated first five dimensions. All plots are related to user 1.

After we computed PCA over the 23 users, we totalled the frequency of occurrence of each feature along the first five dimensions of all users. A feature is included in this count if it had a contribution value over the expected average contribution of all features. The red line in each plot of Figure 4 represents the expected average contribution of all features if their contributions were uniform. This means that the highest possible value of a feature is 115, i.e., it is above a threshold in all five dimensions for every user (5×23). From this sum, we removed from the dataset those features that never contributed (or contributed insufficiently) to any of the first five dimensions, i.e., the dimensions necessary to preserve at least 60% of data variability.

The results in Table 4 show that features F7, F9, and F11 are never used in the PCA analysis; that is, these features do not contribute to explaining the data variability. Furthermore, feature F26 is used only once across the first five dimensions, making its contribution negligible. Hence, it is feasible to remove these features from the PRIDE dataset without losing data representativeness.

**Figure 5:**
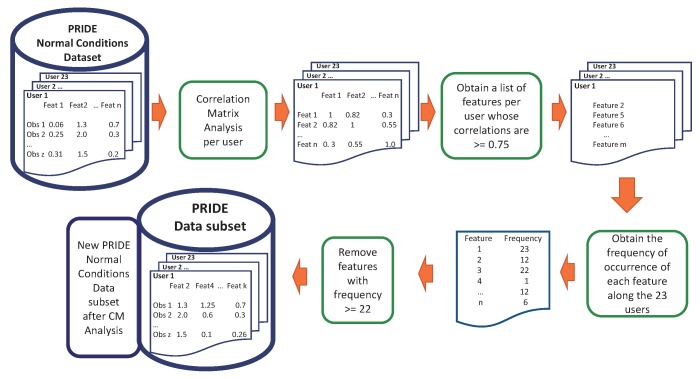
Feature selection process. Phase 1: CM Analysis.

**Figure 6:**
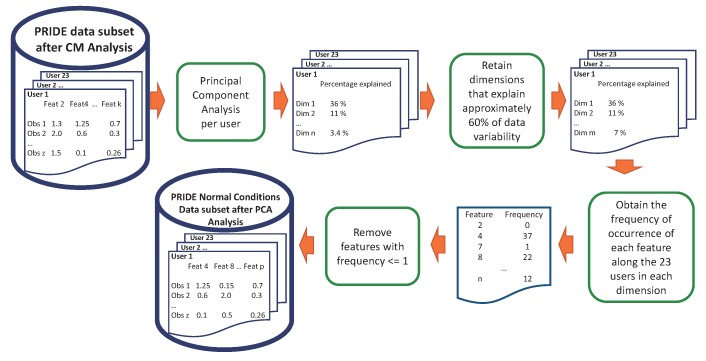
Feature selection process. Phase 2: PCA analysis.

At this point, we have performed a feature selection procedure based on a correlation matrix and Principal Component Analysis in order to reduce the dimension of the PRIDE dataset and thus reduce execution time by keeping a comparable performance of the classifiers. After this procedure, nine attributes in the time-domain were removed without losing data representativeness: five attributes by means of the correlation matrix analysis (F2, F6, F10, F14, and F18) and four attributes by applying Principal Component Analysis (F7, F9, F11, and F26). The complete feature selection process just described is summarised and shown in Figure 5 and Figure 6.

In the next section, we describe the pre-processing of the PRIDE dataset using attributes in the time and frequency domains. Then, a similar procedure for feature selection as the one just described is also presented.

### 3.3. PRIDE Pre-Processing and Feature Selection in the Time/Frequency-Domain

Inspired by [21] and following the aim to obtain features that could describe the behavioural and physiological patterns of a person, several features were calculated in the frequency-domain. Hence, we extended the feature vector presented in Section 3.2 by calculating new frequency-domain attributes; as a result, ten new features in the frequency-domain were derived for each axis of the accelerometer and gyroscope sensors. These features are computed using a non-overlapping one-second time sliding window, similar to the process described in Section 3.2. The accelerometer and gyroscope provide eight data samples every window, since the operation frequency of these sensors is set up at 8 Hz, as recalled from Table 1.

Table 5 shows the new frequency-domain features, computed according to [21,22,23,24]. These attributes come from the signal analysis area and have been widely used to reveal more properties normally not appreciated in the domain of time, to attain a richer view of the observed scenario; the interested reader is referred to these works, for a complete description of the signal processing methods. In total, 90 features in the frequency-domain were obtained. Furthermore, the eight features of non-motion sensors from Table 2 were preserved. Thus, we end up with a 98-dimensional feature vector that combines attributes from both dimensions. For the sake of simplicity to the reader, the final vector is listed in the Appendix. The feature selection process described next is performed over this new feature vector, containing both time and frequency domain features.

In this case, we have also conducted a Principal Component Analysis and a Correlation Matrix analysis on the extended PRIDE dataset with attributes in the time and frequency domains, with the aim of reducing its dimensionality. As in the time-domain case, we first performed the CM process to remove redundant features and then we applied PCA to remove features that do not contribute sufficiently to the principal components, retaining at least 60% of data variability.

We used the same criteria to remove attributes to the dataset as described in Section 3.2; thus, for the sake of simplicity, we only present in the following sections the outcome of the correlation matrix and the Principal Component Analysis.

#### 3.3.1. Correlation Matrix Analysis on the Time/Frequency-Domain Attributes

After performing a correlation matrix analysis on the new feature vector, we were able to remove 11 features from the PRIDE dataset, since their correlation values were consistently above 0.75 in all 23 users. Table 6 shows the features that appeared at least 22 times, thus removed. Since the new vector contains 98 attributes, we only show in the table those attributes removed by the CM analysis, downsizing the vector to 87 attributes.

#### 3.3.2. Principal Component Analysis on the Time/Frequency-Domain Attributes

We run a Principal Component Analysis over every user of the extended PRIDE dataset in order to get rid of those features that do not contribute sufficiently to data variability. After running PCA, we found out that, in this case, the first eight dimensions explain at least 60% of data variability. Then, we added up the frequency of occurrence of each feature along the first eight dimensions of all users. A feature is included in this count if it had a contribution value over the expected average contribution of all features. In this case, the highest possible value of a feature is 184, i.e., it is above a threshold in all eight dimensions for every user (8×23). From this sum, we removed from the dataset those features that never contributed to any of the eight dimensions, i.e., the dimensions necessary to retain at least 60% of data variability.

Results in Table 7 show that features F38, F48, F58 and F98 are never used in the PCA analysis; that is, these features do not contribute to explaining the data variability. Hence, it is feasible to get rid of these features from the PRIDE dataset without losing data representativeness.

In summary, we performed a feature selection procedure based on a correlation matrix and Principal Component Analysis in order to reduce the dimension of the new PRIDE dataset and thus reduce execution time by keeping a comparable performance of the classifiers. After this procedure, fifteen attributes in the time/frequency-domain were removed without losing data representativeness: eleven attributes based on the correlation matrix analysis (F1, F3, F5, F7, F10, F11, F15, F17, F18, F20, and F21) and four attributes after applying a Principal Component Analysis (F38, F48, F58, and F98), resulting in an 83-dimension feature vector.

### 3.4. The Classifiers

We decided to use in our experiments two classifiers, ocSVM and OCKRA. ocSVM is well known in the literature [25] and it was reported by Barrera-Animas et al. in [1] as the best classifier for the personal risk detection problem. On the other hand, OCKRA is a new algorithm proposed in [2] specially designed to improve previous results in the same context and particularly having in mind its implementation in a mobile device. OCKRA is an ensemble of one-class classifiers, based on multiple projections of the dataset according to random subsets of features. Refer to [2] for a detailed description of this new algorithm. For our experiments, we used four different versions of PRIDE, which are:

- Dataset 1. Original dataset as described in [1] based on 26 time-domain features.
- Dataset 2. A subset of Dataset 1, after a feature selection procedure, as described in Section 3.2. Each vector holds 19 attributes.
- Dataset 3. An extended feature vector based on Dataset 1, with new frequency-domain attributes. Each vector comprises 98 attributes.
- Dataset 4. A subset of Dataset 3, after a feature selection procedure, as described in Section 3.3. Each vector holds 83 attributes.

We used the implementation of ocSVM [25] built-in in LibSVM [26] using the radial basis function kernel with default parameter values (γ=0.038 and ν=0.5). Both classifiers, ocSVM and OCKRA, were tested using a five-fold cross-validation, as described in Section 3.2.

In the context of personal risk detection, our intention is to find a classifier that is able to distinguish every possible abnormal behaviour from those that are normal. For that reason, the goal is to build a classifier that maximises true positive classifications (i.e., true abnormal conditions) while minimising false positive ones (i.e., false abnormal or hazardous situations). To evaluate the performance of our classifiers, we compute the Area Under the Curve (AUC) of the true positive detection rate (TPR) versus the false positive detection rate (FPR). This indicator describes the general performance of the classifier for all false positive detection rates.

We only use AUC as the metric to evaluate and compare the classifiers performance since our focus in this work is mainly on the feature selection procedure and the speed-up achieved during training, without sacrificing the classifier performance. We consider AUC a very valuable and robust metric to monitor the overall performance of the classifiers when trained over the four datasets, which all come from the same problem domain, that is, the publicly available PRIDE dataset. For the interested reader, an exhaustive performance comparison between ocSVM and OCKRA over the PRIDE dataset (Dataset 1) is presented in [2]. Therein, in addition to AUC values, Precision–Recall curves and ROC curves (Receiver Operating Characteristic) per user and for the total population are presented, along with several statistical tests.

## 4. Results

Table 8 shows our results regarding the performance of the classifiers based on the AUC metric, where DS-*i* refers to Dataset *i*. In this particular case, averages are acceptable as a quick reference, since the multiple datasets are related to the same problem domain [27]. In general, both classifiers perform better when using datasets DS-1 and DS-2 (i.e., based on time-domain attributes and a subset of it, respectively) than when they are trained using datasets DS-3 and DS-4 (i.e., the extended vector with new attributes in the time/frequency domains and a subset of it, respectively). Based on the performance of both classifiers, we discarded DS-3 and DS-4 for further analysis.

We run a set of Wilcoxon signed-ranks tests to verify whether the two classifiers are statically significantly different or the differences between their performance are random. The first test compares OCKRA against ocSVM run over DS-1, and the second test run over DS-2. According to the results shown in Table 9, we reject the null-hypothesis and decide that OCKRA improves ocSVM with a level of significance α=0.95 and a *p*-value of 0.01. For the second test, Wilcoxon test result is shown in Table 10. We can appreciate that there is also a significant difference between the two classifiers, ocSVM performing better than OCKRA when running over DS-2, this time at a level of significance α=0.90 and a *p* value of 0.049. Although this significant difference is weak (p≈ 0.05), at this point, we cannot conclude that either classifier improves the other in all cases; hence, further analysis is needed.

Regarding execution time, Table 11 shows our results in hh:mm:ss format. We chose the datasets from two users, the ones with more and less number of observations in the PRIDE dataset; that is, users 1 and 17, respectively.

In the case of OCKRA, there is in all instances a reduction in the execution time when training with a subset of the attributes instead of using the full feature vector. The best speed-up is obtained when training user 1 with a subset of attributes in the time-domain (DS-2). In this case, the execution time was approximately 37 min compared to approximately 55.4 min when trained using the full set of attributes (DS-1), which corresponds to a speed-up of 33.1%. For user 17, the attained acceleration is 19.5%. The achieved acceleration is smaller when working with a subset of attributes in the time/frequency domain (DS-3, DS-4); for user 1, 12.2% and for user 17, 15.5%. However, the execution time is much higher, above three hours in the worst case (user 1) compared to 37 min in the previous case. In the case of ocSVM, there is a minimum gain in the execution time when using a subset of attributes against the full feature vector (≤2.2%); however, execution times are much higher in all cases than those reported by OCKRA.

Concerning execution time, it is clear that our best candidate to be implemented on a mobile device is OCKRA using a subset of the time-domain dataset (DS-2). However, as for the performance of the classifiers when using DS-2, we recall from Table 10 that the Wilcoxon test reports a very weak statistical difference, which allows us to take a safe decision when choosing OCKRA as our best candidate without sacrificing performance. Additionally, the gain in speed-up is considerably higher when compared to the execution time reported by ocSVM.

Besides training times, testing and classification times are also important in several real-world applications; however, in our case, these times are very short. We registered the time required for testing by the same users, both classifiers, and the four datasets. We observed seven seconds in the worst case (user 1, OCKRA, DS-3, that is, the extended dataset in the time and frequency domains before feature selection), and less than one second in 25% of the cases. Taking into account that a testing fold contains approximately 94,000 observations for user 1, and 27,000 observations for user 17, the time needed to classify a new object by either OCKRA or ocSVM is negligible.

We can note that, when using the PRIDE dataset based only on time-domain attributes and a subset of it, both classifiers guarantee a good classification performance, which is not the case when using the extended feature vector and a subset of it, as classification performance is notably degraded; however, in the case of OCKRA, the time needed for training can be reduced considerably using the dataset after feature selection. This is a very important fact to consider during the design process, in order to select the more efficient classifier that is to be implemented in a mobile device, assuming less processing and memory resources.

## 5. Discussion

In this work, we built upon previous results reported by Barrera-Animas et al. in [1], in which the authors claimed that it is likely to use PRIDE, a dataset with information drawn from a number of users wearing a device with built-in sensors, to develop a personal risk detection mechanism, and showed that abnormal behaviour could be automatically detected by a one-class classifier. In addition, they showed that OCKRA stands so far as the state-of-the-art classifier in the context of personal risk detection, followed by ocSVM [2]. Our current goal is to derive an efficient classifier to be implemented on mobile devices, as part of a user application for automatic personal risk detection, thus low-consumption of physical resources, such as CPU time and memory, must be taken into account.

First, we decided to extend the PRIDE dataset by adding features in the frequency-domain by transforming current time-domain features on the search to attain a better classification accuracy. Concerning CPU time, we decided to apply feature selection techniques on the PRIDE dataset; in our case, we used correlation matrix and Principal Component Analysis.

We conclude that, in the context of personal risk detection, using the PRIDE dataset based on time-domain attributes and a subset of it should be enough to guarantee a good classification performance. Additionally, OCKRA showed a very important speed-up during the training process when using the dataset after feature selection. Considering the trade-off between classification performance and efficiency, our results support the choice of OCKRA as our best candidate so far for a mobile implementation, using a reduced dataset on the time-domain after a feature selection procedure. By using this subset, OCKRA reported a very important gain on execution time without sacrificing the classifier performance. This result is promising since it can translate into a better user experience, thus reducing the chance to stop using the wearable and its application in the short and mid-term.

Our results are encouraging since they represent the first step towards an efficient classifier well suited for mobile devices and to be implemented as part of a user application coupled with wearable sensors, in order to deal with the problem of timely detecting risk-prone situations experienced by a person.

However, by using our methodology, we acknowledge the possibility that the feature selection process performed on the training dataset could result in removing features that may contribute to the detection of abnormal situations. For example, concerning the UV attribute, the CM analysis kept this feature and the PCA analysis left this variable out, meaning that even though it is not highly correlated to other variables, it does not contribute enough to explain the dataset variability, thus it was removed without losing data representativeness. Therefore, even if we initially thought that UV was a very important feature, PCA tells us that, at least in this dataset, we can safely get rid of it, for the purpose of dimension reduction. The performance of the classifiers was maintained, meaning that the selection process was satisfactory. Nevertheless, it is of great interest to perform the feature selection process on the complete dataset, that is, using the training and testing datasets. Although we believe this can result in overfitting of the dataset, we might be reducing the possibility of leaving out an important feature capable of detecting unseen abnormal behaviours. Therefore, it is clearly a trade-off issue inherent in the feature selection problem for one-class datasets, which is worth further exploring. Indeed, we are currently reviewing other feature selection techniques that allow for a similar reduction on the execution time, but at the same time could achieve a statistically equivalent or better classification performance.

According to Yousef et al. [16,28], feature selection is well studied for two-class classification problems while few methods are proposed for one-class classification ones. Furthermore, two-class feature selection methods may not apply to one-class classification problems because of the use of the two classes during the feature ranking procedure. Thus, this further step becomes challenging, since feature selection is NP-hard according to Yousef et al. Recall from computational complexity theory that a problem is NP-hard if there is an algorithm to solve it that can be transformed into one for solving any NP (non-deterministic polynomial-time) problem. NP-hard is then “at least as hard as any NP-problem”, or even harder. Therefore, additional work must be performed to determine an appropriate feature selection method for the personal risk detection problem.

## Figures and Tables

**Table 1:** Description of sensors in the band.

Sensor	Description	Operation Frequency
Accelerometer	Provides *x*, *y*, and *z* acceleration in g units. 1 g = 9.81 m/s${}^{2}$.	8 Hz
Gyroscope	Provides *x*, *y*, and *z* angular velocity in ${}^{\circ }$/s units.	8 Hz
Distance	Gives the total distance in cm, current speed in cm/s, current pace in ms/m.	1 Hz
Heart Rate	Gives the number of beats per minute.	1 Hz
Pedometer	Delivers the total number of steps the user has accomplished.	1 Hz
Skin Temperature	Gives the current skin temperature of the user in Celsius.	33 mHz
Ultraviolet exposure	Delivers the current ultraviolet radiation exposure intensity.	16 mHz
Calories	Provides total calories burned by the user.	1 Hz

**Table 2:** Feature vector structure.

Feature Number	Feature Name	Feature Number	Feature Name
1	$\bar{x}$ Gyroscope Accelerometer *x*-axis	14	*s* Accelerometer *x*-axis
2	*s* Gyroscope Accelerometer *x*-axis	15	$\bar{x}$ Accelerometer *y*-axis
3	$\bar{x}$ Gyroscope Accelerometer *y*-axis	16	*s* Accelerometer *y*-axis
4	*s* Gyroscope Accelerometer *y*-axis	17	$\bar{x}$ Accelerometer *z*-axis
5	$\bar{x}$ Gyroscope Accelerometer *z*-axis	18	*s* Accelerometer *z*-axis
6	*s* Gyroscope Accelerometer *z*-axis	19	Heart Rate
7	$\bar{x}$ Gyroscope Angular Velocity *x*-axis	20	Skin Temperature
8	*s* Gyroscope Angular Velocity *x*-axis	21	Pace
9	$\bar{x}$ Gyroscope Angular Velocity *y*-axis	22	Speed
10	*s* Gyroscope Angular Velocity *y*-axis	23	Ultraviolet
11	$\bar{x}$ Gyroscope Angular Velocity *z*-axis	24	Δ Pedometer
12	*s* Gyroscope Angular Velocity *z*-axis	25	Δ Distance
13	$\bar{x}$ Accelerometer *x*-axis	26	Δ Calories

**Table 3:** Results of the correlation matrix analysis for feature selection in the PRIDE dataset.

Feature Number	Frequency	Feature Name	Feature Number	Frequency	Feature Name
F1	9	$\bar{x}$ Gyro Accel *x*	**F14**	**22**	**s Accel** *x*
**F2**	**23**	**s Gyro Accel** *x*	F15	15	$\bar{x}$ Accel *y*
F3	8	$\bar{x}$ Gyro Accel *y*	F16	21	*s* Accel *y*
F4	19	*s* Gyro Accel *y*	F17	8	$\bar{x}$ Accel *z*
F5	15	$\bar{x}$ Gyro Accel *z*	**F18**	**23**	**s Accel** *z*
**F6**	**23**	**s Gyro Accel** *z*	F19	0	Heart Rate
F7	0	$\bar{x}$ Gyro Ang Vel *x*	F20	0	Skin Temperature
F8	2	*s* Gyro Ang Vel *x*	F21	3	Δ Pedometer
F9	0	$\bar{x}$ Gyro Ang Vel *y*	F22	13	Δ Distance
**F10**	**22**	**s Gyro Ang Vel** *y*	F23	2	Speed
F11	0	$\bar{x}$ Gyro Ang Vel *z*	F24	0	Pace
F12	10	*s* Gyro Ang Vel *z*	F25	1	Δ Calories
F13	14	$\bar{x}$ Accel *x*	F26	0	Ultraviolet

**Table 4:** Principal Component Analysis results for feature selection in the PRIDE dataset.

Feature Number	Frequency	Feature Name	Feature Number	Frequency	Feature Name
F1	52	$\bar{x}$ Gyro Accel *x*	F16	37	*s* Accel *y*
F3	31	$\bar{x}$ Gyro Accel *y*	F17	53	$\bar{x}$ Accel *z*
F4	37	*s* Gyro Accel *y*	F19	18	Heart Rate
F5	53	$\bar{x}$ Gyro Accel *z*	F20	16	Skin Temperature
**F7**	**0**	**$\bar{x}$ Gyro Ang Vel** *x*	F21	29	Δ Pedometer
F8	28	*s* Gyro Ang Vel *x*	F22	29	Δ Distance
**F9**	**0**	**$\bar{x}$ Gyro Ang Vel** *y*	F23	31	Speed
**F11**	**0**	**$\bar{x}$ Gyro Ang Vel** *z*	F24	29	Pace
F12	26	*s* Gyro Ang Vel *z*	F25	15	Δ Calories
F13	52	$\bar{x}$ Accel *x*	**F26**	**1**	**Ultraviolet**
F15	58	$\bar{x}$ Accel *y*			

**Table 5:** New frequency-domain attributes.

Feature Name	Feature Formula
FFT energy	$\sum_{i=1}^{(\frac{n}{2})+1}x{\left[i\right]}^{2}$
FFT mean energy	$\frac{1}{n}\times \sum_{i=1}^{n}x_{i}$
FFT STD energy	$(\frac{1}{n}\times \sum_{i=1}^{n}x_{i}\left((\right|x_{i}-\frac{1}{n}\times \sum_{i=1}^{n}x_{i}{\left|\right)}^{2}{))}^{2}$
Peak power	max $\frac{P(w_{i})}{\sum_{i}P\left(w_{i}\right)}$
Peak DFT bin	$max_{ith}$ (n×Fs/N)
Peak magnitude	max |fft(x)|
Entropy	$\sum_{j=1}^{(\frac{n}{2})+1}\frac{|x_{i}|}{\sum_{i=1}^{(\frac{n}{2})+1}x{\left[i\right]}^{2}}\times log\left(\frac{|x_{i}|}{\sum_{i=1}^{(\frac{n}{2})+1}x{\left[i\right]}^{2}}\right)$
Spectral Entropy	$-\sum_{i=1}^{n}\frac{P(w_{i})}{\sum_{i}P\left(w_{i}\right)}\times ln\left(\frac{P(w_{i})}{\sum_{i}P\left(w_{i}\right)}\right)$
Peak Frequency	max (n×Fs/N)
Peak energy	max $\sum_{i=1}^{(\frac{n}{2})+1}x{\left[i\right]}^{2}$

Note: FFT stands for Fast Fourier Transform; STD stands for Standard Deviation; DFT stands for Discrete Fourier Transform.

**Table 6:** Results of the correlation matrix analysis for feature selection in the extended PRIDE dataset. Only removed features are shown.

Feature Number	Frequency	Feature Name
F1	23	Energy GyroSensor *x*Accel
F3	23	Standard Deviation Energy GyroSensor *x*Accel
F5	23	Peak DFT Bin GyroSensor *x*Accel
F7	23	Peak Magnitude GyroSensor *x*Accel
F10	23	Peak Energy GyroSensor *x*Accel
F11	22	Energy GyroSensor *y*Accel
F15	23	Peak DFT Bin GyroSensor *y*Accel
F17	23	Peak Magnitude GyroSensor *y*Accel
F18	22	Entropy GyroSensor *y*Accel
F20	23	Peak Energy GyroSensor *y*Accel
F21	23	Energy GyroSensor *z*Accel

**Table 7:** Principal Component Analysis results for feature selection in the extended PRIDE dataset. Only removed features are shown.

Feature Number	Frequency	Feature Name
F38	0	Entropy GyroSensor *x*AngVel
F48	0	Entropy GyroSensor *y*AngVel
F58	0	Entropy GyroSensor *z*AngVel
F98	0	Ultraviolet

**Table 8:** ocSVM and OCKRA performance based on the AUC with different datasets.

User	ocSVM				OCKRA			
	DS-1	DS-2	DS-3	DS-4	DS-1	DS-2	DS-3	DS-4
User 1	97.3	97.3	79.1	78.5	98.8	95.5	78.0	81.0
User 2	94.5	94.3	82.2	81.6	95.7	92.0	85.5	82.9
User 3	87.4	87.2	74.5	73.9	91.2	84.1	82.4	81.9
User 4	83.9	82.1	57.7	57.1	88.2	83.6	61.4	61.0
User 5	80.8	80.8	65.7	65.8	90.2	71.5	68.8	61.9
User 6	96.1	96.1	81.8	81.8	98.2	97.4	87.9	82.3
User 7	69.4	68.1	64.9	64.1	79.2	76.9	65.6	59.5
User 8	93.8	94.0	73.2	71.6	92.4	86.8	77.6	72.8
User 9	95.3	95.5	76.4	75.6	92.7	89.3	81.5	77.8
User 10	94.0	94.3	70.0	69.8	93.7	91.5	69.3	71.9
User 11	93.4	93.8	66.5	66.1	90.9	79.4	69.9	66.8
User 12	74.6	73.4	73.2	73.3	80.3	77.6	71.4	69.4
User 13	75.8	73.4	74.1	73.4	80.5	76.0	70.7	69.2
User 14	78.0	78.2	63.0	62.9	81.9	79.0	66.8	65.1
User 15	93.8	94.4	71.5	70.8	94.5	89.9	77.4	69.3
User 16	83.2	83.0	73.6	73.2	87.9	84.3	73.0	73.8
User 17	98.1	99.0	82.5	82.1	98.0	84.1	81.6	81.9
User 18	89.1	89.0	77.0	77.0	86.9	75.9	70.6	72.5
User 19	89.4	90.0	64.7	64.2	89.6	86.3	64.5	61.8
User 20	90.5	90.2	78.0	77.8	92.2	88.2	79.8	73.6
User 21	98.4	98.4	89.5	89.4	97.9	94.2	87.2	88.3
User 22	78.3	77.8	70.8	70.1	79.2	77.4	70.2	71.5
User 23	53.0	52.6	63.3	62.9	68.9	64.2	72.3	60.9
**Average**	**86.44**	**86.2**	**72.8**	**72.3**	**89.1**	**83.7**	**74.5**	**72.0**

**Table 9:** Wilcoxon signed-ranks test comparison between AUC obtained respectively by ocSVM and OCKRA classifiers when using the DS-1 dataset.

Comparison	*R* ^+^	*R* ^−^	Hypothesis (α=0.05)	*p*-Value
OCKRA vs. ocSVM	221.0	55.0	Rejected	0.010793

**Table 10:** Wilcoxon signed-ranks test comparison between AUC obtained respectively by ocSVM and OCKRA classifiers when using the DS-2 dataset.

Comparison	*R* ^+^	*R* ^−^	Hypothesis (α=0.10)	*p*-Value
ocSVM vs. OCKRA	202.0	74.0	Rejected	0.04979

**Table 11:** Execution time required by the classifier training phase using different datasets. The G column indicates the gain in percentage when using a subset against the full feature vector. Experiments were performed using an Intel core i7-6600U (Mountain View, CA, USA) at 2.60–2.81 GHz and 16 GB RAM.

Domain	Dataset	Dimension	ocSVM				OCKRA			
			User 1	G	User 17	G	User 1	G	User 17	G
Time	DS-1	full	21:14:59		02:39:36		00:55:23		00:04:52	
	DS-2	subset	21:07:07	0.6%	02:38:28	0.7%	00:37:01	33.1%	00:03:55	19.5%
Time+Freq	DS-3	full	19:31:21		01:56:13		03:37:53		00:20:52	
	DS-4	subset	19:05:31	2.2%	01:55:05	0.9%	03:11:17	12.2%	00:17:37	15.5%

## Abbreviations

The following abbreviations are used in this manuscript:

ACDS	Anomaly conditions dataset
AUC	Area under the curve
CM	Correlation matrix
FPR	False positive detection rate
LibSVM	Support vector machine library
NCDS	Normal conditions dataset
OCKRA	One-class K-means with randomly projected features algorithm
ocSVM	One-class support vector machine
PCA	Principal component analysis
PRIDE	Personal risk detection
ROC	Receiver operating characteristic
TPR	True positive detection rate
NP-hard	Non-deterministic polynomial-time hard

## Appendix A

**Table A1:** Time/frequency-domain feature vector part 1.

Feature Number	Feature Name
1	Energy Gyroscope *x*-Axis
2	Mean Energy Gyroscope *x*-Axis
3	Standard Deviation of Energy Gyroscope *x*-Axis
4	Peak Power Gyroscope *x*-Axis
5	Peak DFT Bin Gyroscope *x*-Axis
6	Spectral Entropy Gyroscope *x*-Axis
7	Peak Magnitude Gyroscope *x*-Axis
8	Entropy Gyroscope *x*-Axis
9	Peak Frequency Gyroscope *x*-Axis
10	Peak Energy Gyroscope *x*-Axis
11	Energy Gyroscope *y*-Axis
12	Mean Energy Gyroscope *y*-Axis
13	Standard Deviation of Energy Gyroscope *y*-Axis
14	Peak Power Gyroscope *y*-Axis
15	Peak DFT Bin Gyroscope *y*-Axis
16	Spectral Entropy Gyroscope *y*-Axis
17	Peak Magnitude Gyroscope *y*-Axis
18	Entropy Gyroscope *y*-Axis
19	Peak Frequency Gyroscope *y*-Axis
20	Peak Energy Gyroscope *y*-Axis
21	Energy Gyroscope *z*-Axis
22	Mean Energy Gyroscope *z*-Axis
23	Standard Deviation of Energy Gyroscope *z*-Axis
24	Peak Power Gyroscope *z*-Axis
25	Peak DFT Bin Gyroscope *z*-Axis
26	Spectral Entropy Gyroscope *z*-Axis
27	Peak Magnitude Gyroscope *z*-Axis
28	Entropy Gyroscope *z*-Axis
29	Peak Frequency Gyroscope *z*-Axis
30	Peak Energy Gyroscope *z*-Axis
31	Energy Gyroscope Angular Velocity *x*-Axis
32	Mean Energy Gyroscope Angular Velocity *x*-Axis
33	Standard Deviation of Energy Gyroscope Angular Velocity *x*-Axis
34	Peak Power Gyroscope Angular Velocity *x*-Axis
35	Peak DFT Bin Gyroscope Angular Velocity *x*-Axis
36	Spectral Entropy Gyroscope Angular Velocity *x*-Axis
37	Peak Magnitude Gyroscope Angular Velocity *x*-Axis
38	Entropy Gyroscope Angular Velocity *x*-Axis
39	Peak Frequency Gyroscope Angular Velocity *x*-Axis
40	Peak Energy Gyroscope Angular Velocity *x*-Axis
41	Energy Gyroscope Angular Velocity *y*-Axis
42	Mean Energy Gyroscope Angular Velocity *y*-Axis
43	Standard Deviation of Energy Gyroscope Angular Velocity *y*-Axis
44	Peak Power Gyroscope Angular Velocity *y*-Axis
45	Peak DFT Bin Gyroscope Angular Velocity *y*-Axis
46	Spectral Entropy Gyroscope Angular Velocity *y*-Axis
47	Peak Magnitude Gyroscope Angular Velocity *y*-Axis
48	Entropy Gyroscope Angular Velocity *y*-Axis
49	Peak Frequency Gyroscope Angular Velocity *y*-Axis
50	Peak Energy Gyroscope Angular Velocity *y*-Axis

**Table A2:** Time-/Frequency-domain feature vector part 2.

Feature Number	Feature Name
51	Energy Gyroscope Angular Velocity *z*-Axis
52	Mean Energy Gyroscope Angular Velocity *z*-Axis
53	Standard Deviation of Energy Gyroscope Angular Velocity *z*-Axis
54	Peak Power Gyroscope Angular Velocity *z*-Axis
55	Peak DFT Bin Gyroscope Angular Velocity *z*-Axis
56	Spectral Entropy Gyroscope Angular Velocity *z*-Axis
57	Peak Magnitude Gyroscope Angular Velocity *z*-Axis
58	Entropy Gyroscope Angular Velocity *z*-Axis
59	Peak Frequency Gyroscope Angular Velocity *z*-Axis
60	Peak Energy Gyroscope Angular Velocity *z*-Axis
61	Energy Accelerometer *x*-Axis
62	Mean Energy Accelerometer *x*-Axis
63	Standard Deviation of Energy Accelerometer *x*-Axis
64	Peak Power Accelerometer *x*-Axis
65	Peak DFT Bin Accelerometer *x*-Axis
66	Spectral Entropy Accelerometer *x*-Axis
67	Peak Magnitude Accelerometer *x*-Axis
68	Entropy Accelerometer *x*-Axis
69	Peak Frequency Accelerometer *x*-Axis
70	Peak Energy Accelerometer *x*-Axis
71	Energy Accelerometer *y*-Axis
72	Mean Energy Accelerometer *y*-Axis
73	Standard Deviation of Energy Accelerometer *y*-Axis
74	Peak Power Accelerometer *y*-Axis
75	Peak DFT Bin Accelerometer *y*-Axis
76	Spectral Entropy Accelerometer *y*-Axis
77	Peak Magnitude Accelerometer *y*-Axis
78	Entropy Accelerometer *y*-Axis
79	Peak Frequency Accelerometer *y*-Axis
80	Peak Energy Accelerometer *y*-Axis
81	Energy Accelerometer *z*-Axis
82	Mean Energy Accelerometer *z*-Axis
83	Standard Deviation of Energy Accelerometer *z*-Axis
84	Peak Power Accelerometer *z*-Axis
85	Peak DFT Bin Accelerometer *z*-Axis
86	Spectral Entropy Accelerometer *z*-Axis
87	Peak Magnitude Accelerometer *z*-Axis
88	Entropy Accelerometer *z*-Axis
89	Peak Frequency Accelerometer *z*-Axis
90	Peak Energy Accelerometer *z*-Axis
91	Heart Rate
92	Skin Temperature
93	Δ Pedometer
94	Δ Distance
95	Speed
96	Pace
97	Δ Calories
98	Ultraviolet
